# Common and Rare Radiological Findings of Pulmonary and Thoracic Hydatid Cysts

**DOI:** 10.5152/eurasianjmed.2026.251325

**Published:** 2026-04-15

**Authors:** Yener Aydın, Fatma Durmaz, Ali Bilal Ulaş, Mesut Özgökçe

**Affiliations:** 1 Department of Thoracic Surgery, Atatürk University, Erzurum, Türkiye; 2 Department of Radiology, Van Yüzüncü Yıl University, Van, Türkiye

**Keywords:** Diagnosis, extrapulmonary, hydatid disease, pulmonary, radiology

## Abstract

Clinical findings of pulmonary hydatid cysts (HCs) are generally nonspecific, with lower serological positivity than hepatic cases; therefore, radiological imaging serves as the primary diagnostic tool. Intact cysts on chest radiography present as well-defined, round or oval, homogeneous opacities. Computed tomography remains the gold standard for assessing intact and ruptured cysts, as it delineates internal architecture, membrane detachment, calcification, and relationships with adjacent structures. Magnetic resonance imaging offers complementary value, especially for mediastinal and chest wall cysts, owing to its superior soft-tissue contrast. Characteristic radiological signs after cyst rupture further aid diagnostic assessment. The “air crescent sign,” “water lily sign,” and “serpent sign” carry specificity close to pathognomonic value. Additionally, findings such as the “double dome arch sign” and “air bubble sign” aid in diagnosis. This review aims to provide a comprehensive examination of the radiological findings of pulmonary and thoracic HCs, highlighting the common and rare pathognomonic radiological signs reported in the literature.

**Main Points**

- The diagnosis of pulmonary hydatid cysts (HCs) is generally established radiologically.
- Plain radiography is usually helpful in diagnosing intact cysts; however, careful evaluation is required in women due to breast tissue, and in cases with costophrenic sinus and subpulmonary locations.
- In ruptured cases, radiological findings developing due to cyst walls, hydatid fluid, and membranes, such as the “water lily sign,” “double dome arch sign,” and “serpent sign,” can be diagnostic.
- Knowledge of radiological signs seen in pulmonary and thoracic HCs contributes significantly to the diagnosis, especially in complicated cases.

## Introduction

Hydatid cyst (HC), caused by the larva of *Echinococcus granulosus*, settles in the lungs second most frequently after the liver in the human body.^1^^-^^3^ Within the thoracic cavity, in addition to the pulmonary parenchyma, structures such as the pleura, mediastinum, heart, and diaphragm may also be involved, albeit more rarely.^2^ This review aims to systematically examine common and rare radiological findings in pulmonary and extrapulmonary thoracic HCs and to specifically highlight pathognomonic signs.

### Radiological Diagnosis of Intact Cysts

**Figure 1.**
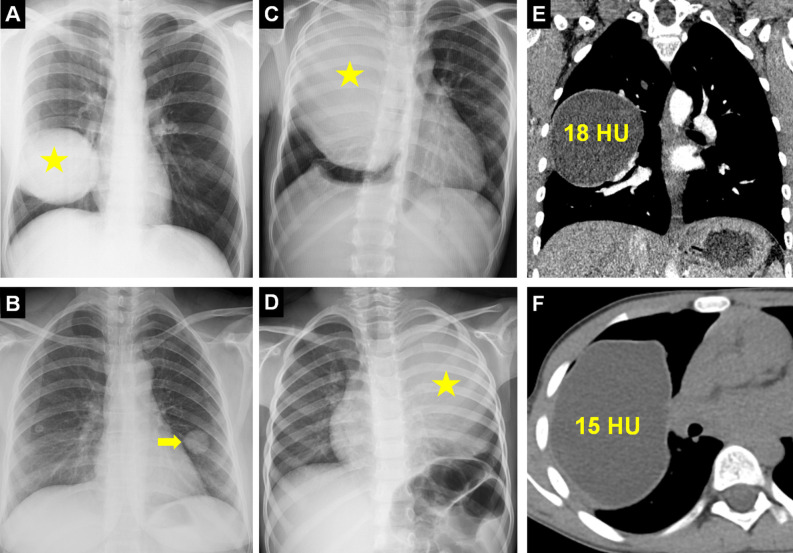
Radiological presentation of intact pulmonary hydatid cysts (HCs). Chest radiograph (A) shows a well-circumscribed, round, and homogeneous opacity (asterisk) due to an intact HC in the right lung. (B) Presentation of an early-detected pulmonary HC as a solitary pulmonary nodule (arrow). (C) Giant HC cases presenting with mediastinal shift in the right lung and (D) in the left lung (asterisks). Confirmation of the cystic content of the lesions via density measurements (Hounsfield Unit - HU) taken on coronal (E) and axial (F) computed tomography images.

Pulmonary HCs are solitary in approximately 70% of cases, and multiple cysts occur in about 30%.^4^ Due to greater lung elasticity in children, pulmonary HCs grow faster and reach larger sizes compared to adults.^5^ Pulmonary HCs may appear as solitary nodules or as giant cysts occupying an entire hemithorax and causing mediastinal shift.^6^^,^^7^Intact pulmonary HCs typically appear on chest radiography as homogeneous, round or oval lesions with smooth margins, often discovered incidentally.^8^ On computed tomography (CT), uncomplicated HCs appear as fluid-attenuation lesions with smooth walls and homogeneous content. Computed tomography density measurements typically demonstrate low Hounsfield Unit (HU) values consistent with fluid, ranging from 3-18 HU in intact cysts, while infected cysts usually exceed 20 HU (Figure 1).^9^ Magnetic resonance imaging (MRI) serves as a valuable adjunct in diagnosing pulmonary HCs, providing detailed assessment of cyst content and wall architecture, particularly in complicated cases where CT findings are inconclusive (Table 1).^10^^,^^11^

**Figure 2.**
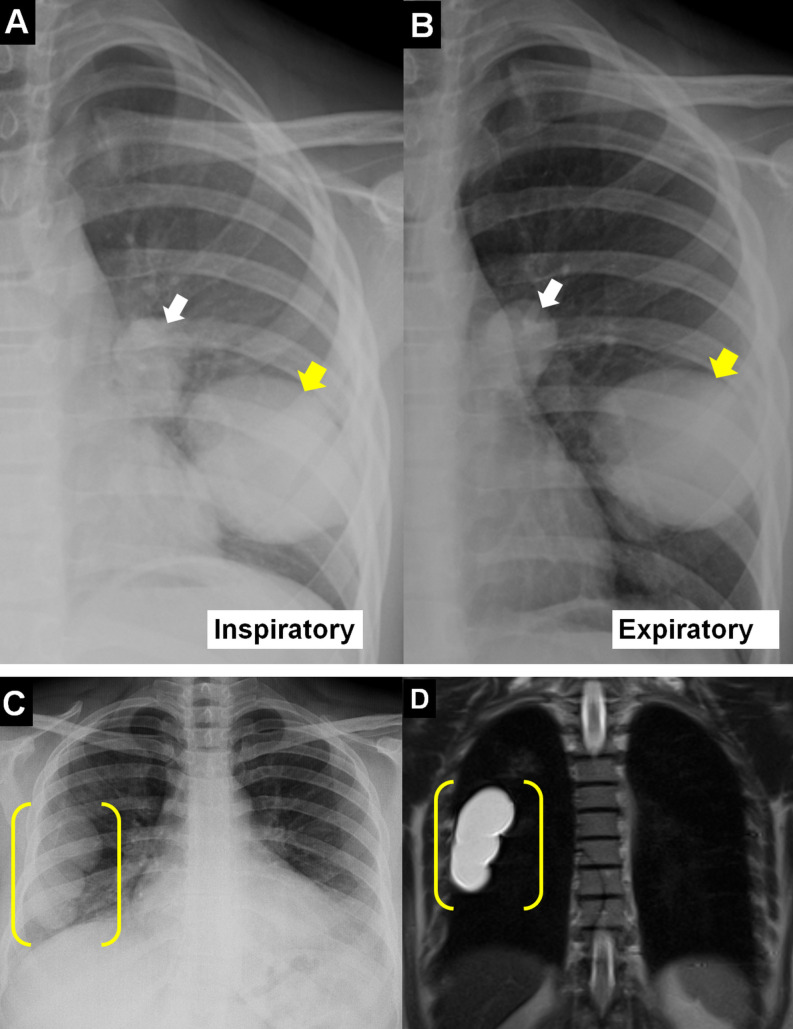
The “escudero-nemerow sign” (arrows), which becomes oval in inspiration (A) and more circular in expiration (B), is seen in intact pulmonary hydatid cysts on chest radiographs. On chest radiograph (C) and magnetic resonance imaging (D), the cyst deviates from round contours and has a conjoined lobulated appearance (frames) due to pressure on surrounding tissue or the developmental process.

Cysts may assume a polycyclic configuration due to pressure from adjacent structures. Adjacent relatively rigid anatomical structures may create a depression or indentation at the contact area, radiologically known as the “notch sign.”^12^ On chest radiography, the shape of the cyst may change radiologically during maximal inspiration and expiration, known as the “Escudero-Nemerow sign” (Figure 2).^13^

**Figure 3.**
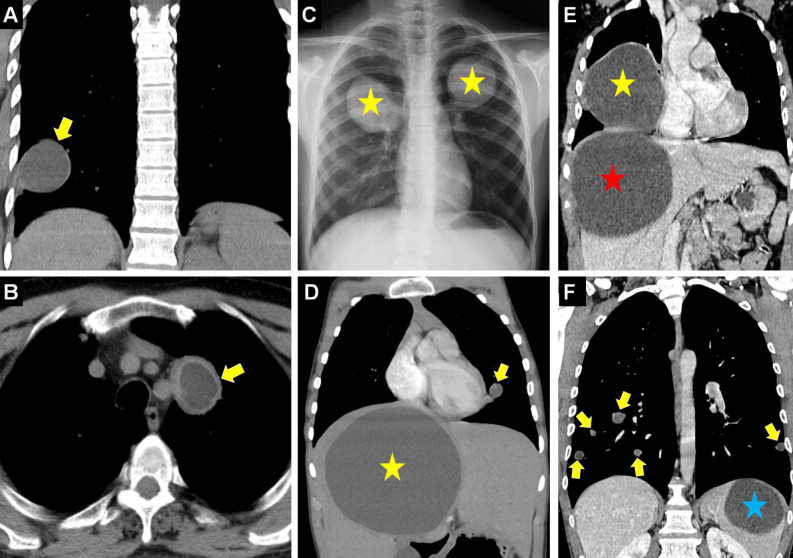
Coronal compued tomography (CT) (A) shows a thin-walled, unilocular, low-density, and uncomplicated hydatid cyst (HC) (arrow). Axial CT (B) shows a unilocular hydatid cyst appearance with a thickened wall structure (arrow). Chest radiograph (C) shows bilateral pulmonary HCs (asterisks). Coronal CT (D) shows simultaneous multiorgan involvement with HCs in the liver (asterisk) and left lung (arrow). Coronal CT (E) shows giant HCs in the liver dome (red-asterisk) and right lung (yellow-asterisk). Coronal CT (F) shows simultaneous HCs in the spleen (asterisk) and bilateral lungs (arrows).

Some cysts may have a thin, imperceptible wall. In infected cysts, a “thick-walled cyst” with a wall thickness of more than 10 mm may be observed.^11^ Pulmonary HCs are seen more frequently in the lower lobes, particularly in the right lower lobe. Generally, it is reported that the right lung is involved in 60% of cases, and bilateral lung involvement is seen in 15% of HCs.^14^^,^^15^ In approximately 35% of cases, concomitant involvement of other organs, primarily the liver, as well as the spleen, kidney, and musculoskeletal system, has been reported along with the lungs.^7^^,^^16^^,^^17^ Multiorgan involvement may aid in radiological recognition, particularly when characteristic signs of hydatid disease are present in multiple organs, potentially enabling earlier diagnosis. However, the diagnostic advantage depends on the specific organs affected and the expertise of the interpreting radiologist (Figure 3).^18^

### Critical Blind Spots in Direct Radiography

**Figure 4.**
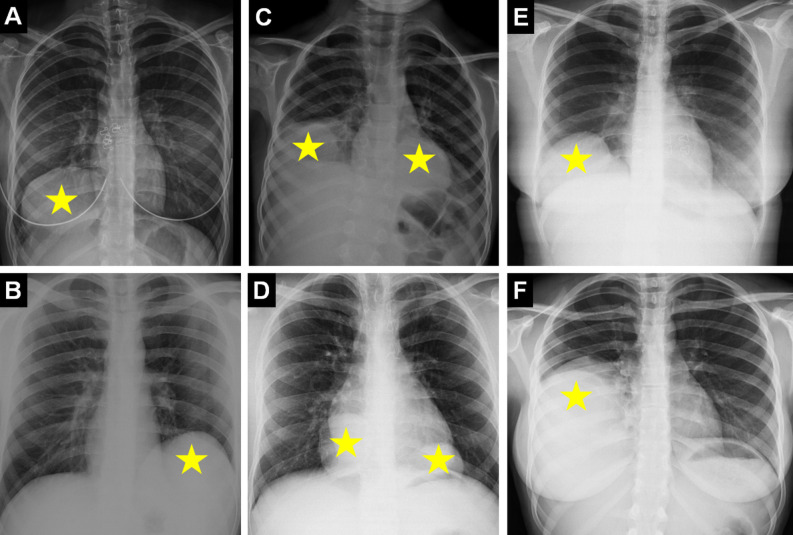
Images of cases with pulmonary hydatid cysts (HCs) located in “blind spots” (regions with high risk of being missed radiographically) on chest radiograph are indicated with asterisks. Hydatid cysts located in the right subpulmonary area (A), left subpulmonary area (B), multifocal in the right subpulmonary and left cardiophrenic angle (C), and in both cardiophrenic angles (D) are seen. The potential of breast tissue opacity to mask pulmonary HCs in female cases is seen on chest radiography (E, F).

The lower lobes are the most common location for HCs. It has been determined that the right lower lobe is involved in 45% and the left lower lobe in 32% of cases.^17^^,^^19^ Hydatid cysts located in the subpulmonary and cardiophrenic sulcus in the lower lobes require careful evaluation on plain radiography. Similarly, in some cases, superimposed with breast tissue, HC may be overlooked on plain radiography (Figure 4).

**Figure 5.**
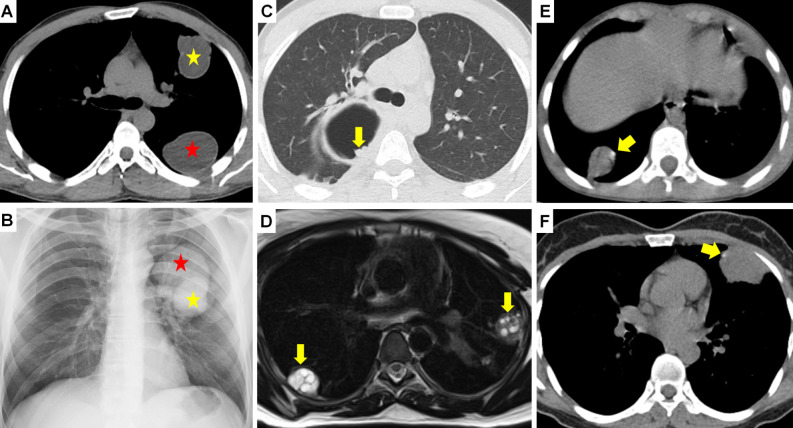
Appearance of 2 simultaneous hydatid cysts (HCs) in the same lung on computed tomography (CT) (A), and the “cyst-in-cyst or silhouette sign” appearance where they overlap and create a silhouette effect on chest radiography (B) (yellow asterisk indicates the anterior cyst, red asterisk indicates the posterior HC). Axial CT (C) shows a ruptured HC and daughter vesicles within the cavity (arrow). T2-weighted MRI (D) shows a multivesicular hyperintense lesion appearance formed by daughter vesicles within HCs in the right and left lungs (arrows). Axial CT shows punctate wall calcifications (arrows) indicating the inactivation or chronic process of the HC in the right (E) and left (F) lungs.

In cases with multiple intact HCs in the same lung, cysts may overlap on plain radiography. A study in the literature naming this situation previously could not be found. This radiological appearance, frequently encountered in endemic regions, was named the “cyst-in-cyst or silhouette sign” by the authors (Figure 5).

### Daughter Vesicle and Calcification

Daughter vesicles are an important biomarker in the diagnosis and prognosis of cystic echinococcosis, indicating that the parasite is still viable and active. The incidence of daughter vesicles is significantly lower in pulmonary localizations compared to hepatic cysts. Magnetic resonance imaging is more successful in demonstrating the relationship with adjacent soft tissues and cyst content, including detached germinal membranes and daughter cysts. Daughter cysts may show low or high signal intensity depending on their varying contents.^20^

Calcification in pulmonary HCs has been reported at a rate of 0.7%.^7^ The low carbon dioxide content within the lung parenchyma reduces the serum calcium level despite the presence of sufficient phosphorus released by tissue necrosis for calcium accumulation, thereby delaying calcium precipitation in dead or degenerated tissue (Figure 5).^11^^,^^21^^-^^23^

### Contained Cyst Rupture

Contained rupture is characterized by an intact laminated membrane, disruption of the pericyst, and absence of intrabronchial communication.

The “air crescent or meniscus or moon sign” is a finding observed on radiography as a thin, radiolucent crescent at the upper part of the cyst, resulting from air entering between the pericyst and the laminated membrane due to the development of bronchial erosion in HC. This sign suggests that the cyst may rupture soon. However, the crescent or meniscus sign is not specific to the hydatid cyst; it can also be seen similarly in other pathologies such as mycetoma, blood clot, carcinoma, Rasmussen aneurysm, pulmonary gangrene, tuberculosis, and sarcoma.^10^^-^^12^^,^^24^

The “inverse crescent sign,” contrary to the classic crescent sign, is a radiological finding characterized by air accumulation in the shape of a crescent on the posterior aspect of the lesion, resulting from dissection from the posterior of the membranes.^12^^,^^19^^,^^20^

The “signet ring sign” is a characteristic radiological finding indicating impending rupture, arising from air bubbles entering between the pericyst and endocyst in HC.^12^^,^^19^^,^^21^

The “air bubble sign” is a radiological finding defined by the visualization of small intracystic air foci between the pericyst and endocyst. The most likely mechanism is air reaching the space between parasitic membranes as a result of erosion caused by bronchioles in the cyst wall. This finding is observed as sharp-bordered single or multiple small round radiolucent areas, especially at the periphery or any part of the cyst, and suggests an infected HC nearing rupture. The “air bubble sign” is best visualized in the mediastinal window in CT. Furthermore, due to its close resemblance to the “signet ring sign,” it has been evaluated as the same finding by some authors.^12^^,^^19^^,^^21^

**Figure 6.**
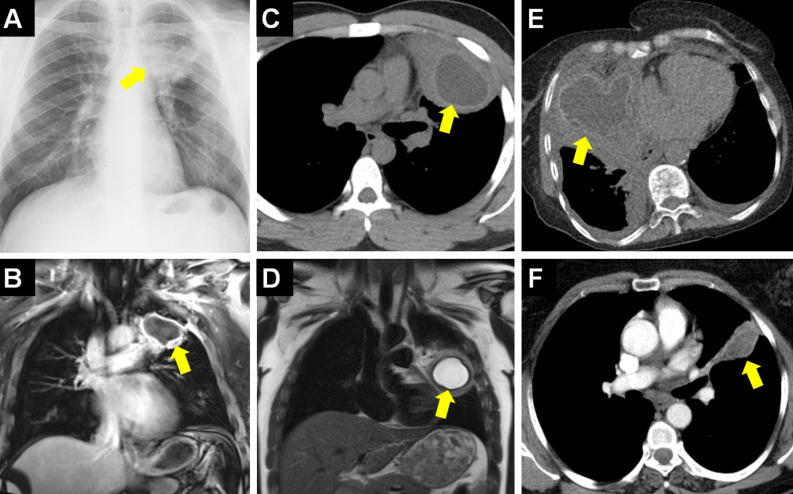
Chest radiograph (A) shows opacity (arrow) belonging to a ruptured and complicated hydatid cyst (HC), and post-contrast magnetic resonance imaging (MRI) (B) of the same patient shows the “ring enhancement sign” (arrow) indicating peripheral contrast enhancement of the cyst wall. The HC is seen as low density on axial computed tomography (CT) (C) and the “hypointense rim sign” in images of the same patient with a hypointense wall on T2-weighted MRI (D) (arrows). Axial CT (E) shows the collapsed endocyst layer (arrow) within the complicated lung parenchyma of a ruptured HC, and in another patient (F), an HC causing atelectasis in the surrounding lung parenchyma (arrow) is seen.

**Figure 7.**
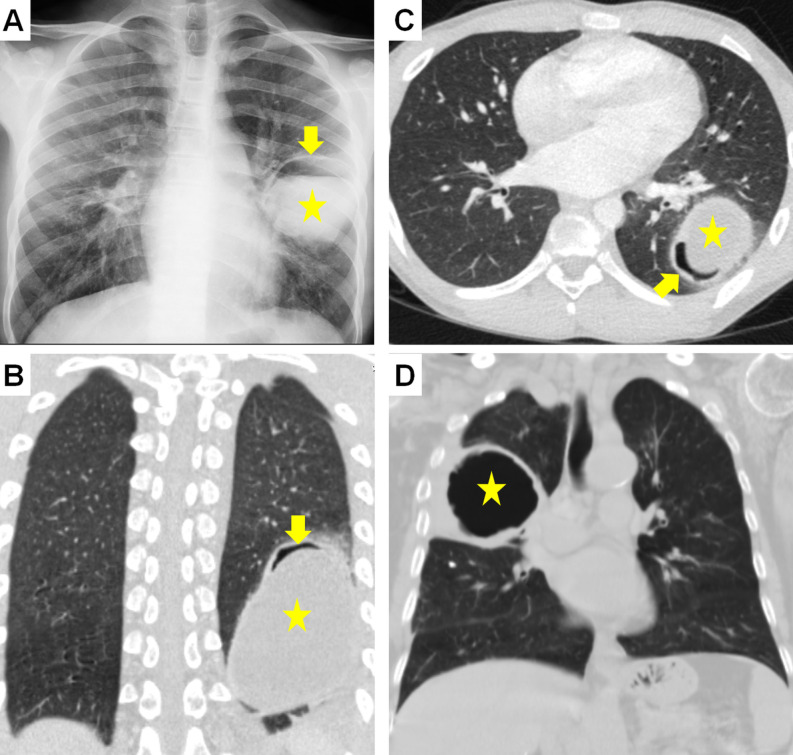
Radiological signs of contained cyst rupture and cyst emptying. In different cases, chest radiograph (A) and coronal parenchymal computed tomography (CT) (B) show the “air crescent or meniscus or moon sign” formed by air entering between the pericyst and laminar membrane (arrow), although the hydatid cyst (HC) is not yet ruptured (asterisks). Axial CT (C) shows the “inverse air crescent sign” (arrow) formed by dissection from the posterior of the HC membranes (asterisk). Coronal CT (D) shows the “empty or dry cyst sign” (asterisk) consisting only of the pericyst wall remaining after the cyst content is completely emptied.

The “ring enhancement sign” is a radiological finding seen in cases of superinfection of HCs, defined as significant thickening of the cyst wall and increased density of the content. Increased contrast enhancement in the form of a ring in the cyst wall on CT and MRI suggests infection and abscess formation. Additionally, the presence of air bubbles or gas within the lesion is among the accompanying indicators of this finding. Magnetic resonance imaging is rarely used in the imaging of pulmonary HCs. Hydatid cyst appears as a capsule with low signal intensity, termed the “hypointense rim sign” on T2-weighted sequences, while appearing hypointense on T1-weighted sequences and hyperintense on T2-weighted sequences (Figures 6 and 7).^19^^9^

### Complete Cyst Rupture

Complete rupture is defined by the presence of findings of a connection with the bronchus. The radiological findings of complete rupture can be listed as follows: “cumbo or double dome arch sign,” “water lily or camolette sign,” “floating membrane sign,” “serpent or snake sign,” “whirl or spin sign,” “empty or dry cyst,” “rising sun sign,” and “mass within the cavity or incarcerated membrane sign.”^20^

In the complete rupture of the endocyst, air enters both inside the endocyst and between the endocyst and pericyst, creating an air-fluid level and parallel air crescents; this characteristic appearance is defined as the “onion peel or cumbo or double dome arch sign.”^7^^,^^25^

The appearance of crumpled membranes floating freely in the cyst fluid at the air-fluid interface as a result of the detachment of the endocyst is called the “water lily or camolette sign.” This appearance arises from the partial or complete collapse of the endocyst and is characterized by undulating membranes floating freely within the cyst fluid. In ruptured HCs, parasitic membranes floating in the fluid and the accompanying air-fluid level are typical findings of this sign. It is relatively rare in pulmonary HCs, seen in approximately 10%-15% of cases.^12^^,^^26^ A similar appearance is also seen to be termed the “floating membrane sign.” The authors believe that it may be more appropriate to term it the “water lily or camolette sign” if the membrane presents a wavy appearance on the hydatid fluid, and the “floating membrane sign” if the membranes are floating within the hydatid fluid.

Parasitic membranes partially collapsing to form serpiginous, twisted structures within the cyst is called the “serpent or snake sign.”^27^ This appearance may sometimes resemble the “dancing hand sign” due to the movement of membranes in the cyst fluid. Following the evacuation or expectoration of cyst fluid, the formation of vortex-like structures by the collapsed membranes within the cyst is defined as the “whirl or spin sign.”^19^

**Figure 8.**
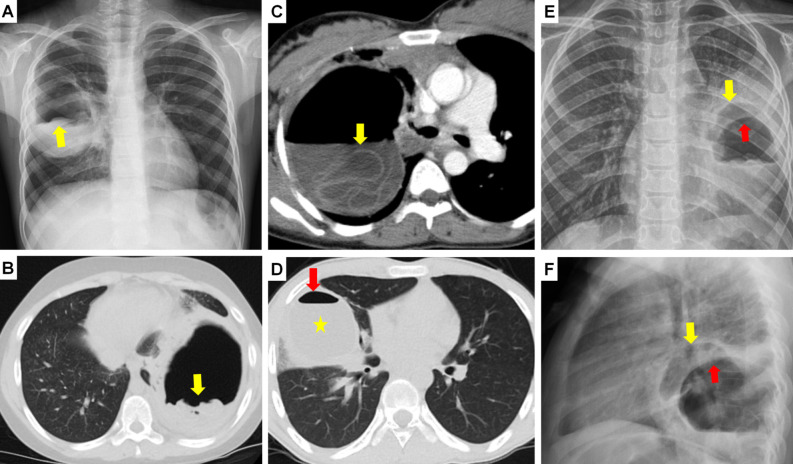
In different cases, chest radiograph (A) and axial computed tomography (CT) (B) show the “water lily or camolette sign” (arrow) formed by the collapsed endocyst floating on the cyst fluid. Axial CT (C) shows the “floating membrane sign” (arrow) formed by the endocyst becoming free after cyst rupture. Axial CT (D) shows the air-fluid level (arrow) formed after a portion of the cyst (asterisk) content emptied into the bronchus. Chest (E) and lateral radiograph (F) of different cases show the “cumbo or double dome arch sign” (arrows) formed by the observation of air-fluid levels within the endocyst and between the endocyst and pericyst, with the endocyst shrinking and rupturing due to increasing air amount.

**Figure 9.**
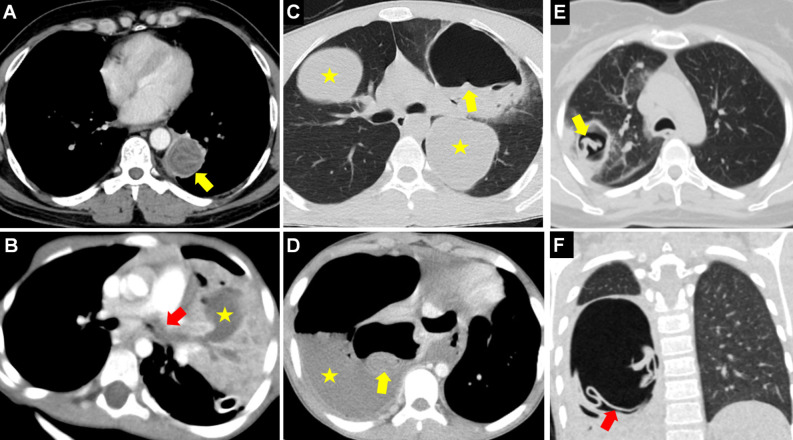
Axial computed tomography (CT) (A) shows the “whirl or spin sign” (arrow) characterized by the germinative membrane (endocyst) remaining free after HC rupture, appearing twisted, contorted, or spirally curled within the cyst fluid. Axial CT (B) shows a hydatid cyst (asterisk) that ruptured directly into the left main bronchus and its branches (arrow). Axial CT (C) shows the “iceberg sign” formed by a large part of the floating membrane in the ruptured cyst remaining under the cyst fluid and only a small part appearing on the surface (arrow). Axial CT (D) shows the “rising sun sign” (arrow) formed by daughter cysts appearing as round radio-opacities at the bottom of the cavity upon rupture of the endocyst, along with accompanying pleural effusion (asterisk). Axial CT (E) shows the “dancing hand sign” (arrow) formed by the membrane becoming free inside after complete rupture of the cyst into the bronchial system. Coronal CT (F) similarly shows the “serpent sign” (arrow) formed by the membrane remaining free after the hydatid fluid is completely emptied.

**Figure 10.**
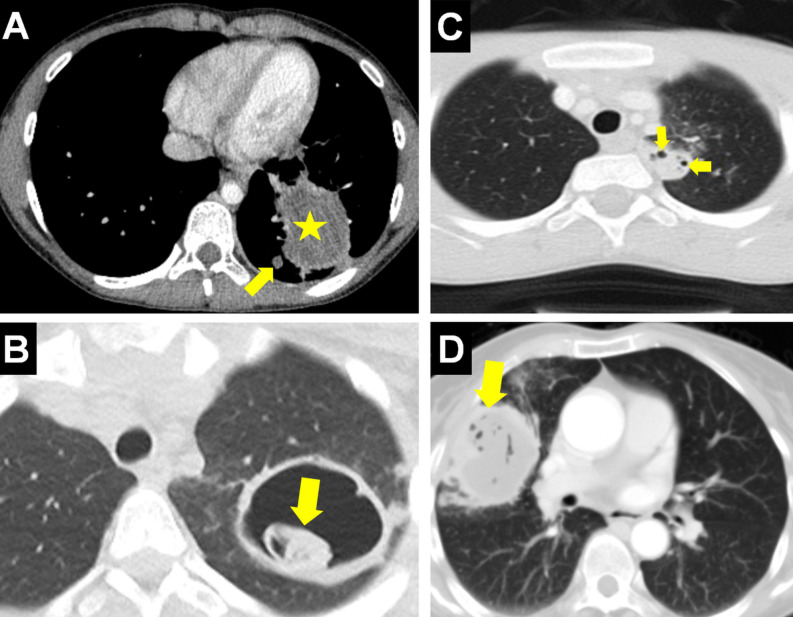
Axial computed tomography (CT) (A) shows a large hydatid cyst (HC) (asterisk) and a smaller satellite HC (arrow) next to it. Axial CT (B) shows the “mass within the cavity or incarcerated membrane sign” (arrow) formed by the remaining solid parts settling at the bottom of the cavity after the cyst fluid is completely emptied by expectoration. Axial CT (C) shows the “signet ring sign” (arrows) formed by an air bubble trapped at the periphery of the cyst between the pericyst and endocyst. Axial CT (D) shows an infected HC containing an “air bubble” (arrow) and ground-glass and mild tree-in-bud appearances in the surrounding parenchyma.

Although a large part of the cystic content is expectorated, the entrapment of some membrane residues in the ruptured cyst cavity or at the opening of the bronchocystic fistula is called the “retained cyst sign.”^22^ The settlement of the detached and crumpled endocyst at the lowest part of the cavity is defined as the “mass within the cavity or incarcerated membrane sign.”^7^^,^^19^ Daughter cysts seen as round radio-opacities at the bottom of the cysts upon rupture of the endocyst form the “rising sun sign” appearance; this finding is rare in pulmonary HC and, when present, is observed as circular shadows among fragmented membranes.^12^^,^^19^ Finally, when all parasitic content is evacuated, and only the host-derived pericyst remains, the empty and air-filled cyst appearance is called the “empty or dry cyst sign.” Although this typical finding facilitates diagnosis on CT, it may not always appear in the classical form in complicated cysts (Figure 8-10).^6^^7^^,^^11^^,^^19^

### Complications Following Hydatid Cyst Rupture and Differential Diagnosis

Pleural necrosis from pressure of peripheral cysts may contribute to rupture into the pleural cavity, which can manifest as effusion, pneumothorax, hydropneumothorax, empyema, pleural thickening, lung collapse, or bronchopleural fistula. This acute presentation, often without prior hepatic or pulmonary HC findings, poses diagnostic challenges. Chest radiographs typically reveal effusion, pneumothorax, hydropneumothorax, or lung opacities, the latter reflecting atelectasis, pneumonia, or ruptured infected cysts.^28^ Besides endocyst rupture, cysts may directly involve the parenchyma, main bronchus, or pleural cavity. Parenchymal rupture can cause consolidation, whereas endobronchial extension may produce centrilobular opacities.

**Figure 11.**
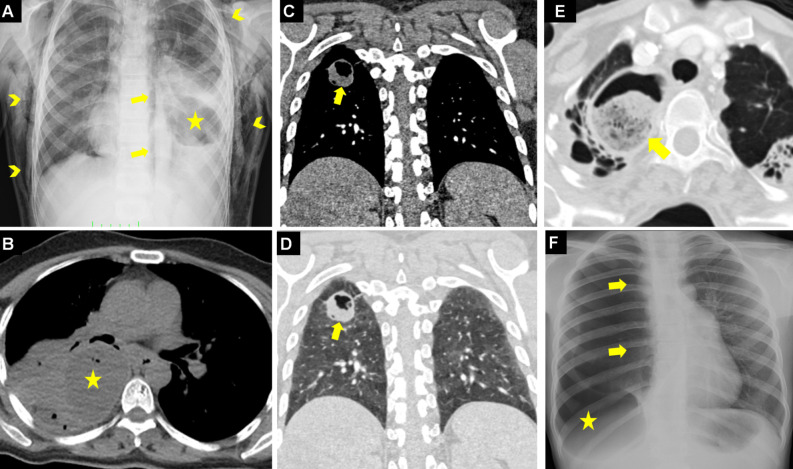
Chest radiograph (A) shows pneumomediastinum (arrows) and diffuse subcutaneous emphysema (arrowheads) developed secondary to a ruptured cyst (asterisk). Axial computed tomography (CT) (B) shows a hydatid cyst (HC) containing air bubbles, requiring differential diagnosis from a slightly high-density abscess. Mediastinal (C) and parenchymal (D) coronal CT of the same patient show the HC (arrows) consistent with a cavitary lesion appearance radiologically developed after the evacuation of cyst content. Computed tomography (E) shows debris (arrow) within a chronic and complicated cyst cavity mimicking an aspergilloma lesion. Chest radiograph (F) shows a life-threatening tension pneumothorax on the right caused by cyst rupture (asterisk), pushing the mediastinum and heart to the opposite side (arrows).

**Figure 12.**
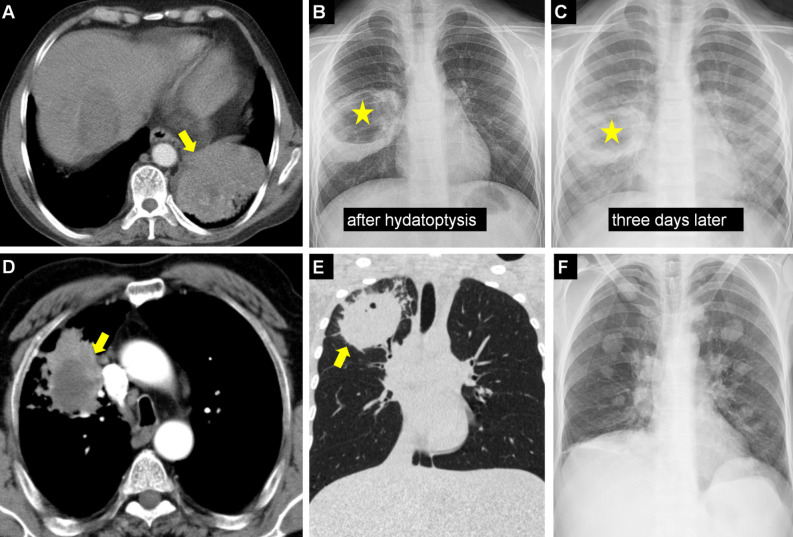
Axial computed tomography (CT) (A) shows a hydatid cyst (arrow) causing a consolidated appearance due to bronchial leakage or inflammation in the surrounding lung parenchyma and histopathologically leading to organizing pneumonia. Chest radiographs of the same case show an allergic reaction due to hydatid fluid expectoration immediately after rupture (B) and dense pneumonic infiltration and cyst cavities (arrow) on the third-day radiograph (C). Axial CT (D) shows a cyst (arrow) located adjacent to the superior vena cava, which can mimic lung cancer due to mass effect. Coronal CT (E) shows a complicated cyst (arrow) with irregular borders and peripheral spicular extensions mimicking lung cancer morphology. Chest radiograph (F) shows an atypical presentation of diffuse, multiple cysts in both lungs, which can be radiologically confused with the appearance of pulmonary metastasis.

**Figure 13.**
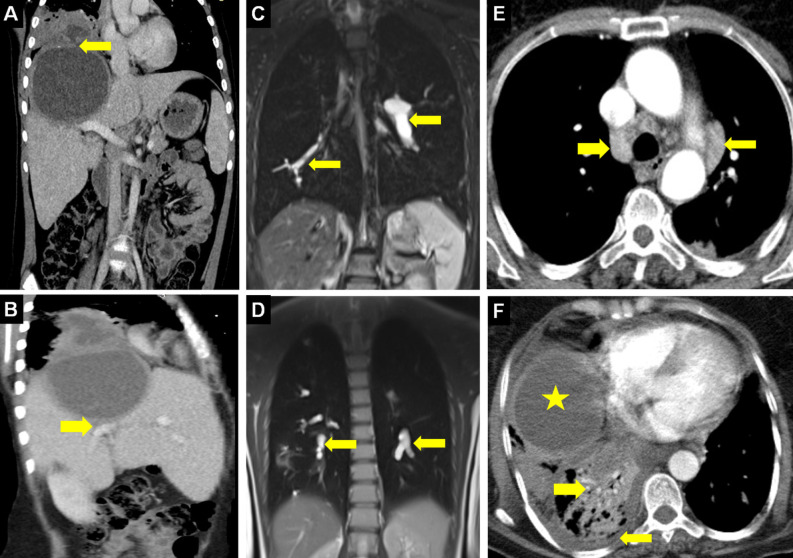
Coronal computed tomography (CT) (A) shows the fistula (arrow) of a hydatid cyst (HC) extending exophytically from the liver to the subdiaphragmatic area, to the diaphragm, and from there to the lung; and multiplanar reformatted sagittal section (B) shows a BBF with small connections to the intrahepatic bile ducts from inferior (arrow). Coronal T2-weighted magnetic resoance imaging (C, D) shows multiple hyperintense defects within pulmonary arteries (arrows) caused by intravascular hydatid content (hydatid embolism). Axial CT (E) shows mediastinal lymphadenopathies (arrows) due to ruptured and infected HC, and in a different case (F), an HC (asterisk) causing pleural effusion and consolidation (arrow) containing dilated air bronchograms (arrow) in the right lung is observed.

Complicated and uncomplicated cysts may be accompanied by atelectasis, bronchiectasis, mediastinal lymphadenopathy, pleural thickening, or effusion. Bronchial obstruction and parenchymal destruction can result in distal bronchiectasis. Differentiating complicated cysts from tumors, hematomas, congenital cysts, or pneumothorax is challenging. Infected cysts with air-fluid levels may resemble lung abscesses, while air-filled cysts emptied of contents can mimic cavitary lesions such as aspergilloma, necrotic malignancies, or tuberculous cavities (Figure 11-13).^29^^-^^32^

### Pulmonary Embolism

Hepatic HCs may occasionally rupture into the inferior vena cava, resulting in recurrent pulmonary embolism, while right-sided cardiac cysts can rupture directly into the pulmonary arteries.^12^^,^^19^ Diagnosis can be made with pulmonary CT angiography showing non-enhancing HCs causing expansion in the lumen of pulmonary arteries. On MRI, the intra-arterial cyst is typical with low signal intensity on T1-weighted images and high signal intensity on T2-weighted images.^6^ Pulmonary artery filling defects of cystic nature, coupled with absent enhancement, help differentiate this entity from thromboembolism and primary or secondary pulmonary artery tumors (Figure 13).^33^^,^^34^

### Bronchobiliary Fistula

Bronchobiliary fistula (BBF) is an abnormal connection between the right bronchial tree and the biliary system and is an extremely rare complication of HC.^22^ Diagnosis of BBF is difficult, and the only specific finding may be biliptysis, which can be in small or large amounts. In terms of diagnostic methods, chest radiography may show lung collapse or pleural effusion. Computed tomography scans can reveal pulmonary, hepatic, or subphrenic collections and parenchymal damage. In these cases, imaging methods such as endoscopic retrograde cholangiopancreatography, hepatobiliary scintigraphy (HIDA scan), and magnetic resonance cholangiopancreatography may be useful (Figure 13).^35^

### Extrapulmonary Thoracic Hydatid Cysts

Extrapulmonary thoracic HCs are very rare and may coexist with lung involvement or be found in isolation.^2^

Cardiac HCs are primarily evaluated with echocardiography and CT, which demonstrate their relationship to cardiac and extracardiac structures. Echocardiography, as a cornerstone modality, provides rapid, non-invasive information on cyst morphology, location, size, and functional impact. Magnetic resonance imaging offers superior evaluation of cyst internal structure, wall features, daughter vesicles, adjacent cardiac relationships, and extracardiac extension.^36^

Diagnosis of diaphragmatic HC is challenging. Computed tomography effectively demonstrates cyst localization and extension but often fails to reveal diaphragmatic defects, whereas MRI provides clearer visualization of both cyst structure and diaphragmatic involvement.^37^

In chest wall HCs, contrast-enhanced CT and MR imaging exhibit characteristic findings similar to involvement in other organs. On CT, chest wall involvement can be observed as a multiloculated mass with daughter cysts. A multiloculated osteolytic lesion developing in the rib may present as an extrapleural soft tissue mass and lead to cortical expansion or destruction. These primary rib lesions grow slowly and may extend into adjacent structures such as the vertebrae, pleura, or subcutaneous soft tissues. While extraosseous cysts may show calcification, intraosseous cysts rarely calcify. The absence of osteoporosis and sclerosis, preservation of intervertebral disc spaces, and paraspinal extension are typical features of spinal HCs.^20^^,^^38^

**Figure 14.**
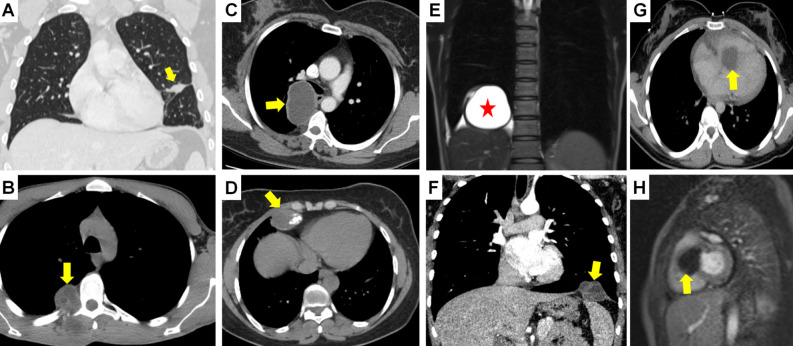
Computed tomography (CT) (A) shows a hydatid cyst (HC) mimicking a phantom tumor located at the fissure level in the left lung (arrow); CT (B) shows an extrapulmonary multivesicular HC (arrow) located in chest wall structures (muscle/bone) outside the lung parenchyma. Computed tomography of different cases shows HCs located in the posterior (C) and anterior mediastinum (D) (arrows). Calcification is seen in the anterior mediastinal HC. Magnetic resonance imaging (E) shows an HC (asterisk) starting from the right subdiaphragmatic area and involving the diaphragm, and CT (F) shows a multivesicular HC (arrow) with primary localization in the left hemidiaphragm. Computed tomography (G) and MRI (H) sections of the same case show a cardiac HC (arrows) protruding from the interventricular septum towards the right ventricle.

Imaging findings of mediastinal HCs may vary, presenting as unilocular, multilocular, complicated, or calcified cysts (Figure 14).^12^^,^^38^

## Conclusion

The recognition and differentiation of radiological findings in pulmonary HCs present significant diagnostic challenges, particularly in distinguishing ruptured cysts from other pathologies such as lung abscesses, aspergillomas, and cavitary tuberculosis. The air crescent sign, while relatively specific for impending rupture, is not pathognomonic and requires careful correlation with clinical presentation and patient epidemiology. Similarly, the water lily sign and serpent sign, though highly characteristic of ruptured cysts, may be intermittently visualized depending on the timing of imaging relative to cyst rupture and bronchial communication. The diagnostic approach should therefore integrate clinical findings, serological markers when available, and radiological pattern recognition.

Furthermore, awareness of common diagnostic pitfalls such as missed diagnosis of subpulmonary and cardiophrenic cysts, confusion with breast tissue opacity in female patients, and misinterpretation of complicated cysts as malignancy is essential for accurate diagnosis. Computed tomography remains the gold standard for characterizing both intact and complicated cysts, while MRI provides valuable complementary information for assessing cyst-wall characteristics and relationships with critical structures. The specific radiological findings have direct clinical implications: identification of daughter cysts or thick-walled cysts suggests parasite viability and guides treatment selection; detection of rupture with bronchial communication necessitates urgent intervention; and recognition of complications such as empyema, bronchopleural fistula, or pulmonary artery involvement determines the urgency and complexity of surgical or medical management.

## Figures and Tables

**Table 1.** Radiological Findings Associated with Pulmonary Hydatid Cyst

**Findings in Intact Cysts**	**Complete Cyst Rupture**
Cyst with smooth walls	Cumbo sign or double dome arch sign
Thin wall unilocular cyst	Water lily sign or camolette sign
Escudero-Nemerow sign	Floating membrane sign
Solitary pulmonary nodule	Serpent sign or snake sign
Multiple cysts	Whirl or spin sign
Silhouette sign	Empty or dry cyst
Bilateral pulmonary cysts	Rising sun sign
Giant cyst	Mass within the cavity or incarcerated membrane
Involvement with extrapulmonary organs	
Mediastinal shift	**Complications Following Rupture**
Polycyclic and bilobed appearance	Pneumothorax
	Pleural effusion
**Obscured Cysts on Chest X‑Ray**	Subcutaneous–mediastinal emphysema
Subpulmonary Region	Atelectasis
Cardio-phrenic sulcus	Abscess formation
Breast tissue in women	Air-fluid level
	Mediastinal-hilar lymph node
**Other Finding**	Pneumonia
Daughter cyst	Aspergilloma
Calcification	Malignancy mimic
	Pulmonary embolism
**Contained Cyst Rupture**	Bronchobiliary fistula
Air crescent or meniscus or moon sign	
Inverse crescent sign	
Signet ring sign	
Air bubble sign	
Ring enhancement sign	
Thick wall unilocular cyst	

Comprehensive summary of radiological findings associated with pulmonary hydatid cysts (HCs). This table lists characteristic radiological findings observed in intact, contained rupture, and completely ruptured HCs. Intact cysts typically demonstrate well-defined borders, smooth or thin walls, and homogeneous low-attenuation content on computed tomography. Findings obscured on chest radiography include cysts in the subpulmonary region, cardiophrenic sulcus, or those obscured by breast tissue in women. Contained rupture findings include the air crescent sign (air between pericyst and laminated membrane), inverse crescent sign, signet ring sign, and air bubble sign. Complete rupture findings include the water lily/camolette sign, cumbo/double dome arch sign, whirl/spin sign, serpent/snake sign, rising sun sign, and empty/dry cyst sign.

## Data Availability

The data that support the findings of this study are available on request from the corresponding author.
